# Rapid Degradation of Hfq-Free RyhB in *Yersinia pestis* by PNPase Independent of Putative Ribonucleolytic Complexes

**DOI:** 10.1155/2014/798918

**Published:** 2014-04-10

**Authors:** Zhongliang Deng, Zizhong Liu, Yujing Bi, Xiaoyi Wang, Dongsheng Zhou, Ruifu Yang, Yanping Han

**Affiliations:** ^1^Department of Sanitary Inspection, School of Public Health, University of South China, Hengyang, Hunan 421001, China; ^2^State Key Laboratory of Pathogen and Biosecurity, Beijing Institute of Microbiology and Epidemiology, Beijing 100071, China

## Abstract

The RNA chaperone Hfq in bacteria stabilizes sRNAs by protecting them from the attack of ribonucleases. Upon release from Hfq, sRNAs are preferably degraded by PNPase. PNPase usually forms multienzyme ribonucleolytic complexes with endoribonuclease E and/or RNA helicase RhlB to facilitate the degradation of the structured RNA. However, whether PNPase activity on Hfq-free sRNAs is associated with the assembly of RNase E or RhlB has yet to be determined. Here we examined the roles of the main endoribonucleases, exoribonucleases, and ancillary RNA-modifying enzymes in the degradation of *Y. pestis* RyhB in the absence of Hfq. Expectedly, the transcript levels of both RyhB1 and RyhB2 increase only after inactivating PNPase, which confirms the importance of PNPase in sRNA degradation. By contrast, the signal of RyhB becomes barely perceptible after inactivating of RNase III, which may be explained by the increase in PNPase levels resulting from the exemption of *pnp* mRNA from RNase III processing. No significant changes are observed in RyhB stability after deletion of either the PNPase-binding domain of RNase E or *rhlB*. Therefore, PNPase acts as a major enzyme of RyhB degradation independent of PNPase-containing RNase E and RhlB assembly in the absence of Hfq.

## 1. Introduction

Small regulatory RNAs (sRNAs) function as posttranscriptional regulators by altering translation or stability of the target mRNA, which increases their applicability in different physiological processes in bacteria [1]. The RNA chaperone Hfq is hypothesized to facilitate the access of sRNAs to their mRNA targets and stabilize sRNAs by protecting them from the attack of RNase E [2]. Given that the increasing amount of available information on sRNA-induced mRNA decay is accumulating [3–6], the sRNA degradation processes and RNases that catalyze such activities must be investigated. The multienzyme assembly of RNA degradosome is important for mRNA decay and processing in* Escherichia coli*. RNase E and polynucleotide phosphorylase (PNPase) are two major components of the RNA degradation process [7, 8]. RNase E is also responsible for the rapid degradation of sRNAs and competes with Hfq in accessing the same RNA sequences [9–11]. Hfq recruits RNase E by directly interacting with the RhlB-recognition region, which is hypothesized to cause the coupled cleavage of mRNA and sRNA [6, 12]. PNPase plays the protective role in the RNase E-dependent degradation in the presence of Hfq [13, 14]. Recent studies show that Hfq has a limited access to RNAs under wild-type conditions considering the dynamic interactions of Hfq with sRNAs [15–17]. A transient Hfq-free state of sRNAs may also be observed. A recent study shows that sRNAs are preferably degraded by the major exoribonuclease PNPase upon release from Hfq [14]. PNPase usually cooperates with RNase E in RNA degradation complexes [18]. RNA helicase RhlB usually facilitates RNA degradation by manipulating RNA structure and remodeling ribonucleoprotein complexes in the presence or absence of RNase E [19]. However, the relationship between the PNPase activity in Hfq-free sRNAs and RNA degradation complexes remains unknown.

The well-characterized sRNA RyhB was used as a model sRNA for this study. RyhB is an Hfq-binding sRNA that maintains iron homeostasis in bacteria [20, 21]. Besides Hfq, RyhB also becomes very stable when the overall mRNA transcription is stalled in* E. coli* [6]. Two RyhB homologs possessing the conserved core and* rho* sequences in* E. coli* [20] have also been characterized in* S. typhimurium* [22]. RyhB1 and RyhB2 are upregulated in the infected lungs of mice upon intranasal inoculation of* Yersinia pestis*, which indicates that they may serve as important functions during* Y. pestis* pathogenesis. The stability of RyhB1 and RyhB2 is differentially Hfq-dependent in* Y. pestis* grown under nutrient-limiting conditions [23]. This study constructs single or combined* hfq* mutant strains that lack various RNases or ancillary enzymes and monitors the expression level and degradation speeds of RyhB to investigate the effect of these enzymes on the degradation of Hfq-free RyhB.

## 2. Materials and Methods

### 2.1. Bacterial Strains and Growth Conditions

All strains are derivatives of* Y. pestis* strain 201, a newly established biovar, the* Microtus* [24].  Table 1 shows the bacterial strains that are used in this study. Except for the RNase E mutants, all mutant strains were constructed by replacing the entire gene with an antibiotic cassette via *λ*-Red homologous recombination. RNase E is essential for viability in bacteria, but deleting the C-terminal half (CTH) of this enzyme is not lethal [26]. The CTH after the 910th containing putative PNPase-binding site (1190-1221aa corresponding to 1021-1061aa in* E. coli* RNase E) [26] was deleted and designated as *rne*_910_. Bacteria were grown to midexponential phase (*A*_620_ ≈ 1.0) in BHI medium at 26°C. Iron depletion was induced by adding 100 *μ*M 2′,2′-dipyridyl (DIP) for 20 min. Antibiotics were added when needed at the following concentrations: 34 *μ*g/mL chloramphenicol, 50 *μ*g/mL kanamycin, 100 *μ*g/mL ampicillin, 20 *μ*g/mL gentamicin, and 20 *μ*g/mL streptomycin.

### 2.2. RNA Extraction and Northern Blotting Analysis

Pure bacterial cultures were mixed with RNAprotect Bacteria Reagent (Qiagen) to minimize RNA degradation. The total RNA was then extracted from* Y. pestis* using TRIzol Reagent (Invitrogen). Northern blotting analysis was performed by using a DIG Northern Starter Kit (Roche) according to the manufacturer's protocol described by Beckmann et al. [27]. RNA samples (3 *μ*g) were denatured at 70°C for 5 min, separated on 6% polyacrylamide-7M urea gel, and transferred onto Hybond N^+^ membranes (GE) via electroblotting. The membranes were UV-crosslinked and prehybridized for 1 hr, and 3′-end DIG-labeled RNA oligonucleotides were added. The membranes were then hybridized overnight at 68°C in a DIG Easy Hyb. RNA was immunologically detected and scanned according to the instructions. Multiple exposures to X-ray film were taken to achieve the desired signal strength.

### 2.3. RNA Half-Life Determination

Bacteria grown to exponential phase were treated with 250 *μ*g/mL rifampicin for RNA half-life determination. Culture samples were collected at 0, 5, 10, 20, 30, and 60 min and were subject to RNA extraction and Northern blotting. Films were scanned and RNA band intensity was measured using Quantity One software. The intensities were plotted and RNA half-lives were calculated using the slope from each plot.

### 2.4. Quantitative RT-PCR

Total RNA was isolated from different* Y. pestis* strains grown to exponential growth phase (OD_620_ = 1.2) in BHI by using Trizol Reagent (Invitrogen). DNA contaminants were removed by using DNA-free Kit (Ambion), and the cDNA was converted by using random hexamer primers with the Superscript II system (Invitrogen). Real-time PCR was performed in duplicate for each RNA preparation by using the LightCycler system (Roche) with an appropriate dilution of cDNA as a template. Negative controls without reverse transcriptase enzyme were included in all experiments. Relative quantitative analysis across different cDNA templates was performed by using LightCycler 480 software (Bio-Rad) with the 16S rDNA as the normalized gene.

## 3. Results and Discussion

### 3.1. Influence of RNases and Ancillary RNA-Modifying Enzymes on the Regulation of Hfq-Free RyhB

BHI was selected as the growth medium for bacterial culture because some mutants that were constructed in this study experienced a slow growth upon inoculation into TMH medium, which pose a challenge to our experiments.

**Figure 1:**
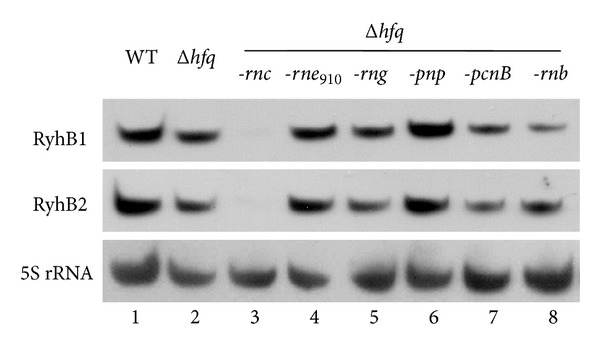
Effects of RNases and an ancillary RNA-modifying enzyme on the transcriptional level of* Y. pestis* RyhB1 and RyhB2 in the Δ*hfq* background. RyhB1 and RyhB2 were detected by Northern blotting using 5 *μ*g of total RNA extracted from* Y. pestis* grown to exponential phase in BHI medium upon treatment with 100 *μ*M DIP treatment for 20 min. 5S rRNA was used as a negative control. Lanes 1–8 represent WT (lane 1),* hfq* mutant (lane 2), double mutants lacking* hfq*, and another gene encoding either endoribonucleases (RNase E_910_, RNase G) (lanes 4 and 5), exoribonucleases (RNase III, PNPase, and RNase II) (lanes 3, 6, and 8), or ancillary RNA-modifying enzyme (polyA polymerase) (lane 7).

The expressions of RyhB1 and RyhB2 were monitored in multiple* hfq* mutants that lacked major RNases or ancillary RNA-modifying enzymes to validate the influence of endoribonucleases, exoribonucleases, and ancillary RNA-modifying enzymes on RyhB regulation in* Y. pestis* without Hfq (Figure 1). The expression levels of RyhB1 and RyhB2 slightly increased (~1.8-fold) upon the deletion of PNPase, but no obvious changes were observed in the RNase E truncate and deletion strains of RNase G (*rng*), RNase II (*rnb*), or polyA polymerase (*pcnB*). In contrast, RyhB was rarely detected in the double mutants that lacked Hfq and RNase III (*rnc*).

**Figure 2:**
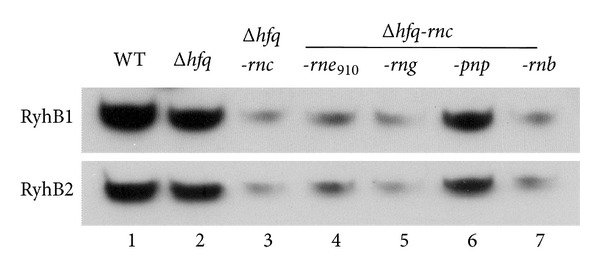
Effects of various ribonucleases on the transcriptional level of RyhB1 and RyhB2 upon inactivation of Hfq and RNase III. RyhB1 and RyhB2 were detected by Northern blotting using 5 *μ*g of total RNA extracted from* Y. pestis* grown to exponential phase in BHI medium upon treatment with 100 *μ*M DIP treatment for 20 min. Lanes 1–7 represent WT (lane 1),* hfq* mutant (lane 2),* hfq-rnc* double mutants (lane 3) and triple mutants lacking* hfq*,* rnc*, and another gene encoding RNase E_910_ (lane 4), RNase G (lane 5), PNPase (lane 6), or RNase II (lane 7).

The* rne* (910-1221aa),* rng*,* pnp*, and* rnb* genes were deleted from the double deletion mutant that lacked Hfq and RNase III to determine which RNases account for the degradation of RyhB1 and RyhB2, respectively (Figure 2). RyhB in the* hfq-rnc-pnp* mutant reached a similar amount of that in the* hfq* mutant, which indicates that PNPase was the main contributor in the degradation of Hfq-free RyhB [14].

The degradation of Hfq-free RyhB by PNPase tends to occur in stationary phase rather than exponential phase in* E. coli* [14]. However, the inactivation of PNPase in this study increased the RyhB levels in* Y. pestis* grown to exponential phase. Therefore, PNPase may degrade the Hfq-free RyhB in different growth-phase-dependent manners in* E. coli *and in* Y. pestis*. However, such discrepancy may also be due to the different sample timing that was used in these two experiments. It would be helpful to make it clear if more time-point samplings are included in these experiments.

### 3.2. The RNase-III-Inactivation-Induced mRNA Level Increase of PNPase May Be Partially Responsible for the Degradation of Hfq-Free RyhB

**Figure 3:**
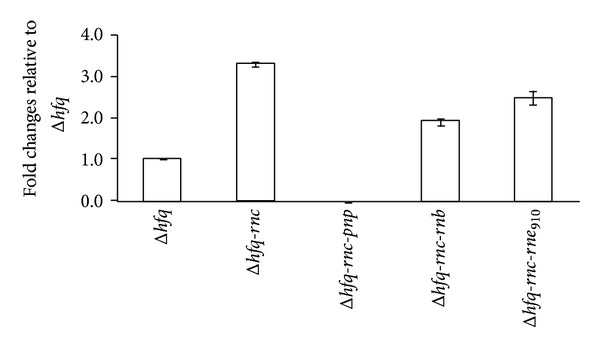
Expression levels of the* pnp* mRNA in multiple mutants of* Y. pestis* by using quantitative PCR. RNA samples were prepared from various mutants lacking Hfq and other ribonucleases grown to exponential phase in BHI medium. The relative abundance of the* pnp* mRNA was accessed by real-time PCR.

Few amounts of* micA* could be also detected in the* hfq-rnc* double mutant of* E. coli* [14]. Andrade et al. explained this phenomenon as an impairment of RNase III activity that was caused by the decreased duplex in the absence of Hfq. However, this impairment cannot explain the obvious difference in RyhB expression between* hfq* and* hfq-rnc* double mutant. RNase III can alter gene expression by cleaving dsRNA or by binding without cleaving RNA [28]. RNase III has been proved to involve in the autoregulation of PNPase in* E. coli* by cleaving the 5′ end of* pnp* mRNA [29]. However, the unprocessed* pnp* mRNA is accumulated and can be translated into polynucleotide phosphorylase in* E. coli rnc* mutant [29]. To determine if the inactivation of RNase III affected the expression of PNPase, quantitative PCR was performed to estimate the relative amounts of* pnp* mRNA in different mutants (Figure 3). The* pnp* gene was upregulated from 1.9- to 3.3-fold in* hfq-rnc* double and triple mutants than in the* hfq* mutant, which further confirmed that PNPase was the main exoribonuclease responsible for the degradation of* Y. pestis* RyhB in the absence of* hfq*. The RNase-III-inactivation-induced upregulation of PNPase could be partially responsible for the decreased expression of RyhB (Figure 2). However, the effects of RNase III on RyhB stability could not be determined through other means.

### 3.3. PNPase Activity on RyhB in the Absence of RNase III Is Dependent on the State of Hfq Binding

**Figure 4:**
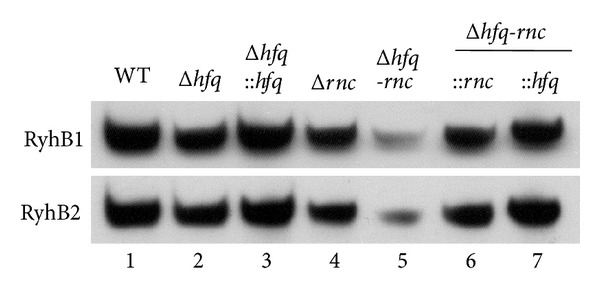
Effects of Hfq and RNase III on the transcriptional level of RyhB1 and RyhB2 in* Y. pestis*. Total RNAs were extracted from WT,* hfq/rnc* single or double mutants, and their complementary strains and then were subject to Northern blotting analysis.

RNase III affects the stability of the Hfq-dependent sRNA, MicA, in* Salmonella* [30]. The expression patterns of single and double mutants of* rnc* and* hfq* were compared via Northern blotting to examine the effects of RNase III and Hfq inactivation on the rapid degradation of RyhB. RyhB was rarely detected after inactivating both RNase III and Hfq. However, the amount of RyhB could reach modest levels in the* rnc* and* hfq* single mutants as well as in the complementary strains that carried the corresponding plasmids. Therefore, the PNPase activity on RyhB in the absence of RNase III depends on the state of Hfq binding (Figure 4). RyhB was rapidly degraded by the increased levels of PNPase in the absence of Hfq because of the RNase III inactivation.

### 3.4. Rapid Degradation of Hfq-Free RyhB by PNPase Is Independent of the PNPase-Containing RNase E or RhlB Assembly

**Figure 5:**
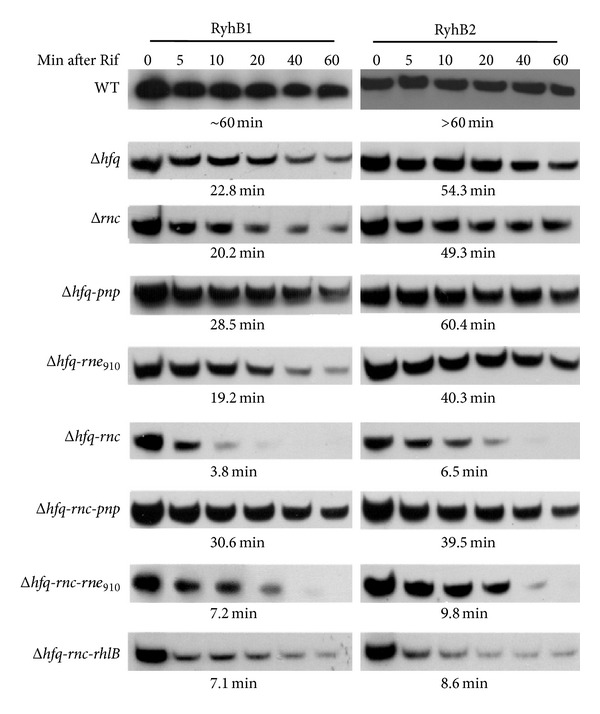
Effects of various RNases or ancillary RNA-modifying enzymes on RyhB stability in Hfq-lacking* Y. pestis.* Various mutants grown to exponential phase were treated with 250 *μ*g/mL of rifampicin. Culture samples were collected at 0, 5, 10, 20, 40, and 60 min and were subject to RNA extraction and Northern blotting, respectively.

RyhB1 was rapidly degraded, but RyhB2 retained its stability in the absence of* Y. pestis hfq* grown in TMH medium [23]. In* Y. pestis hfq* mutant grown in BHI medium, RyhB1 obtained a 22.8 min half-life whereas RyhB2 obtained a 54.3 min half-life (Figure 5). Although the Hfq-dependent stabilities of* Y. pestis* RyhB1 and RyhB2 remained different in this study, RyhB1 showed a significantly higher stability in bacterial cells that were grown in rich media (with *a* > 20 min half-life) than in bacterial cells that were grown in minimal media (with ~8 min half-life). The half-lives of both RyhB1 and RyhB2 exceeded 60 min in a WT strain that was grown exponentially in BHI medium (data not shown), which indicated that the nutrition conditions would influence the stability of* Y. pestis* RyhB in the absence of Hfq.

The half-lives of RyhB in the* hfq-pnp* double mutant were investigated to verify the effects of PNPase on the degradation of Hfq-free RyhB (Figure 5). The stability of RyhB slightly increased in the* hfq-pnp* double mutant rather than in the* hfq* single mutant, which confirmed the role of PNPase in the degradation of Hfq-free RyhB. The* rnc* deletion mutation produced insignificant effects on the stability of RyhB with half-lives of 20.2 min and 49.3 min (Figure 5). However, the 14 min decrease in the half-life of RyhB2 in the* hfq-rne*_910_ double mutant remains unclear. The half-lives of RyhB dramatically reduced to 3.8 min and 6.5 min in the* hfq-rnc* double mutant, whereas the deletion of the* pnp* gene increased the half-life of RyhB to >30 min (Figure 5). Therefore, the RNase-III-induced PNPase increase might be responsible for the RyhB degradation in the absence of Hfq, and the PNPase served as the main enzyme in the degradation of Hfq-free RyhB.

PNPase usually forms multienzyme ribonucleolytic complexes with RNase E and/or RNA helicase RhlB during the degradation of the structured RNA [31, 32]. RNase E serves as a “scaffolding” protein of RNA degradosome that contains the binding sites of three major degradosome components, namely, PNPase, DEAD-box helicase RhlB, and enolase [8, 33]. RhlB facilitates the formation of single stranded RNA, which helps PNPase to engage in the 3′ to 5′ exoribonucleolytic degradation of RNA [15]. PNPase directly interacts with RhlB by forming the transient complex, which is not dependent on the formation of the degradosome [34]. Therefore, this study tries to determine if RNase E degradosome is involved in PNPase activity on Hfq-free RyhB. Given that the deletion of the* rne* gene in the encoding of RNase E is lethal, an* rne* mutant without PNP-binding domain was constructed in this study to produce an RNase E protein that was unassociated with PNPase.

The Northern blotting analysis revealed that the mutation of* rne* and* rhlB* had *a* > 7 min half-life in the* hfq-rnc* mutant, but its stability was substantially lower than that upon PNPase inactivation (Figure 5). Therefore, the PNPase-containing degradosome or exosome plays minor roles in Hfq-free RyhB decay, and PNPase might be involved in these processes by itself or through other unknown mechanisms. Therefore, the degradation of Hfq-free sRNAs is far more complex than what was previously expected. An extended analysis should be performed to check if these results could be applied to other sRNAs.

## Figures and Tables

**Table 1:** Bacterial strains used in this study.

Strains	Relevant characteristics	Sources or reference
WT	Wild-type strain 201	[24]
Δ*hfq *	*hf* *q* ^−^	[25]
Δ*hfq::hfq *	*hf* *q* ^−^::pACYC184-*hfq*	[25]
Δ*rnc *	*rn* *c* ^−^	This study
Δ*hfq- * *rne*_910_	*hf* *q* ^−^ *rn* *e*_910-1061aa_^−^	This study
Δ*hfq-rng *	*hf* *q* ^−^ *rn* *g* ^−^	This study
Δ*hfq-rnc *	*hf* *q* ^−^ *rn* *c* ^−^	This study
Δ*hfq-pnp *	*hf* *q* ^−^ *pn* *p* ^−^	This study
Δ*hfq-rnb *	*hf* *q* ^−^ *rn* *b* ^−^	This study
Δ*hfq-rnr *	*hf* *q* ^−^ *rn* *r* ^−^	This study
Δ*hfq-pcnB *	*hf* *q* ^−^ *pc* *nB* ^−^	This study
Δ*hfq-rhlB *	*hf* *q* ^−^ *rh* *lB* ^−^	This study
Δ*hfq-rnc- * *rne*_910_	*hf* *q* ^−^ *rn* *c* ^−^ *rn* *e*_910-1061aa_^−^	This study
Δ*hfq-rnc-rng *	*hf* *q* ^−^ *rn* *c* ^−^ *rn* *g* ^−^	This study
Δ*hfq-rnc-pnp *	*hf* *q* ^−^ *rn* *c* ^−^ *pn* *p* ^−^	This study
Δ*hfq-rnc-rnb *	*hf* *q* ^−^ *rn* *c* ^−^ *rn* *b* ^−^	This study
Δ*hfq-rnc-rnr *	*hf* *q* ^−^ *rn* *c* ^−^ *rn* *r* ^−^	This study
Δ*hfq-rnc-rhlB *	*hf* *q* ^−^ *rn* *c* ^−^ *rh* *lB* ^−^	This study
